# Surgery of highly eloquent gliomas primarily assessed as non-resectable: risks and benefits in a cohort study

**DOI:** 10.1186/1471-2407-13-51

**Published:** 2013-02-02

**Authors:** Sandro M Krieg, Lea Schnurbus, Ehab Shiban, Doris Droese, Thomas Obermueller, Niels Buchmann, Jens Gempt, Bernhard Meyer, Florian Ringel

**Affiliations:** 1 Department of Neurosurgery, Klinikum rechts der Isar, Technische Universität München, Ismaninger Str. 22, 81675, Munich, Germany; 2 Department of Anesthesiology, Klinikum rechts der Isar, Technische Universität München, Ismaninger Str. 22, 81675, Munich, Germany

**Keywords:** Language, Eloquent tumor, Rolandic region, Glioma, Neuromonitoring.

## Abstract

**Background:**

Today, the treatment of choice for high- and low-grade gliomas requires primarily surgical resection to achieve the best survival and quality of life. Nevertheless, many gliomas within highly eloquent cortical regions, e.g., insula, rolandic, and left perisylvian cortex, still do not undergo surgery because of the impending risk of surgery-related deficits at some centers. However, pre and intraoperative brain mapping, intraoperative neuromonitoring (IOM), and awake surgery increase safety, which allows resection of most of these tumors with a considerably low rate of postoperatively new deficits.

**Methods:**

Between 2006 and 2012, we resected 47 out of 51 supratentorial gliomas (92%), which were primarily evaluated to be non-resectable during previous presentation at another neurosurgical department. Out of these, 25 were glioblastomas WHO grade IV (53%), 14 were anaplastic astrocytomas WHO grade III (30%), 7 were diffuse astrocytomas WHO grade II (15%), and one was a pilocytic astrocytoma WHO grade I (2%). All data, including pre and intraoperative brain mapping and monitoring (IOM) by motor evoked potentials (MEPs) were reviewed and related to the postoperative outcome.

**Results:**

Awake surgery was performed in 8 cases (17%). IOM was required in 38 cases (81%) and was stable in 18 cases (47%), whereas MEPs changed the surgical strategy in 10 cases (26%). Thereby, gross total resection was achieved in 35 cases (74%). Postoperatively, 17 of 47 patients (36%) had a new motor or language deficit, which remained permanent in 8.5% (4 patients). Progression-free follow-up was 11.3 months (range: 2 weeks – 64.5 months) and median survival was 14.8 months (range: 4 weeks – 20.5 months). Median Karnofsky Performance Scale was 85 before and 80 after surgery).

**Conclusions:**

In specialized centers, most highly eloquent gliomas are eligible for surgical resection with an acceptable rate of surgery-related deficits; therefore, they should be referred to specialized centers.

## Background

For the treatment of high- and low-grade gliomas, surgery is an important part of a multimodal therapy [1-4]. Surgical tumor reduction has been shown to have a impact on survival and quality of life and, thus, has to be as extensive as possible [1,3-5]. Nonetheless, many gliomas within highly eloquent regions, especially within the insula, rolandic region, and the perisylvian cortex of the dominant hemisphere, still frequently undergo limited debulking or biopsy attributable to the supposed risk of surgery-related deficits [6-9]. Resection of such highly eloquent gliomas always involves a compromise between the extent of resection and the preservation of motor or language function. To achieve both goals, neurosurgeons use multiple modalities to examine, visualize, and monitor anatomy and function presurgically and during resection [10-15]. By carefully choosing a multimodal setup including preoperative mapping of motor and language function using navigated transcranial magnetic stimulation (nTMS), intraoperative cortical and subcortical mapping using direct cortical stimulation (DCS), intraoperative neuromonitoring (IOM), and awake surgery, we can increase safety and, therefore, allow resection of most such tumors with an acceptable rate of postoperative new deficits [14-23]. Although the literature and data on eloquent glioma surgery are broad, no studies or subgroup analyses are at hand that analyzed the actual functional outcome and oncological benefit of surgery in patients initially diagnosed as inoperable. Thus, we present this retrospective analysis and evaluated all cases that presented to our department for a second opinion. Neurological course, preoperative nTMS, intraoperative DCS mapping, and IOM data were reviewed and related to new postoperative deficits and postoperative imaging. Moreover, clinical outcomes were assessed during follow-up.

## Methods

### Patients

Between 2006 and 2012, we resected 47 out of 51 supratentorial gliomas, which were primarily judged to be non-resectable during prior consultation at another neurosurgical department. These departments were European university departments or at least of university level concerning the range and numbers of surgeries. Four patients with glioma of the basal ganglia did not undergo surgical resection but stereotactic biopsy. During this period between 2006 and 2012, 498 patients underwent surgery of intracranial gliomas in our department.

Decision for surgery was made during an interdisciplinary conference including neurosurgeons, neuro-oncologists, neuroradiologists, neuropathologists, and radiation oncologists in all cases. An overview of all patients is given in Table 1. In 9 out of these 47 cases (19%), the tumor was located within or adjacent to the precentral gyrus, in 15 cases (32%) within the insula, in 7 cases (15%) within the postcentral gyrus, in 3 cases (6%) within the basal ganglia, in 5 cases (11%) within the opercular inferior frontal gyrus, in 5 cases (11%) within the middle superior temporal gyrus, and in 3 cases (6%) within the supramarginal gyrus. Mean tumor diameter was 4.9 ± 2.6 cm (range 0.4 – 11.0 cm). Tumor size was assessed on T2 FLAIR images for WHO grade II and II and on T1 contrast-enhanced images for WHO grade I and IV. A preoperative motor deficit was present in 13 patients (28%). Median Karnofsky performance scale (KPS) was 90 (range 40 – 100%). The mean age was 47 ± 16 years (range 17 – 81 years); 19 patients (40%) were female and 28 (60%) were male. Twenty-seven tumors (59%) were in the dominant hemisphere. Indication for awake surgery was a glioma within the left insular and perisylvian region with sufficient remaining language function to perform an intraoperative object naming and counting task. Out of 47 cases, 25 were glioblastomas WHO grade IV (53%), 14 were anaplastic astrocytomas WHO grade III (30%), 7 were diffuse astrocytomas WHO grade II (15%), and one was a pilocytic astrocytoma WHO grade I (2%). As this report wants to draw attention on the resectability of gliomas per se, we also included this pilocytic astrocytoma in our series because especially these tumors should undergo resection.

**Table 1:** Patient characteristics

**Pt #**	**WHO grade**	**Recurrent tumor**	**Tumor diameter**	**Preop TMZ**	**Preop RTx**	**Preop motor deficit**	**Postop motor deficit**	**Preop language deficit**	**Postop language deficit**	**Preop KPS**	**Postop KPS**	**EOR**	**Awake surgery**
1	III	Y	11.0	Y	Y	N	N	N	N	90	90	GTR	N
2	III	N	5.1	N	N	N	T	N	T	70	60	GTR	Y
3	III	Y	6.8	Y	N	Y	N	N	N	70	70	GTR	N
4	III	Y	10.8	N	N	N	T	N	N	90	70	GTR	N
5	IV	Y	9.2	Y	N	Y	N	N	T	80	80	STR	Y
6	IV	N	4.5	N	N	N	N	N	T	70	70	GTR	Y
7	IV	Y	1.9	Y	Y	N	T	N	N	80	60	STR	N
8	IV	Y	0.4	Y	Y	N	N	N	N	100	100	STR	N
9	IV	Y	4.5	Y	Y	Y	T	N	N	90	90	GTR	N
10	IV	N	5.6	Y	N	N	N	N	N	90	90	GTR	N
11	II	Y	5.1	N	N	N	N	N	N	90	90	GTR	Y
12	III	Y	4.5	Y	N	N	N	N	N	100	100	GTR	N
13	II	N	7.9	Y	N	Y	T	N	T	70	70	GTR	Y
14	II	N	6.1	N	N	N	N	N	N	100	100	GTR	N
15	IV	Y	3.0	Y	Y	N	N	N	N	100	100	GTR	N
16	III	Y	6.4	N	N	N	N	N	N	100	100	GTR	N
17	IV	Y	7.0	Y	Y	N	T	N	N	70	30	GTR	N
18	III	Y	0.7	Y	N	N	T	N	N	100	80	GTR	N
19	III	N	9.3	N	Y	N	N	N	N	100	100	GTR	N
20	II	Y	4.6	Y	N	N	T	N	T	100	90	GTR	Y
21	IV	Y	2.6	Y	Y	N	T	N	N	50	50	GTR	N
22	IV	Y	2.0	Y	Seed	Y	P	N	P	80	70	STR	Y
23	IV	Y	4.9	Y	Y	N	N	N	N	90	80	GTR	N
24	IV	N	6.8	Y	N	Y	N	N	N	70	70	STR	N
25	II	N	5.6	N	N	N	N	N	N	70	70	STR	N
26	IV	Y	2.9	Y	Y	Y	N	N	N	70	70	GTR	N
27	IV	N	4.6	N	N	N	N	N	N	90	90	GTR	N
28	III	Y	4.0	Y	N	Y	P	N	N	90	50	GTR	N
29	IV	Y	6.0	Y	Y	N	N	N	N	80	80	GTR	N
30	III	N	10.0	Y	N	N	N	N	N	90	90	STR	N
31	III	N	5.3	Y	Y	N	N	N	N	90	90	GTR	N
32	IV	N	3.3	N	Y	Y	N	N	N	40	40	STR	N
33	III	N	4.0	Y	N	N	N	N	T	90	90	STR	Y
34	II	Y	1.5	N	N	N	N	N	N	100	100	GTR	N
35	III	Y	1.1	Y	N	N	P	N	N	90	40	GTR	N
36	IV	Y	6.0	N	N	N	N	N	N	90	90	GTR	N
37	IV	Y	1.4	Y	Y	N	T	N	N	100	50	GTR	N
38	III	Y	7.1	Y	N	N	T	N	N	100	60	STR	N
39	I	N	1.5	N	N	Y	N	N	N	50	100	GTR	N
40	IV	Y	2.4	Y	Y	Y	T	N	N	60	50	GTR	N
41	IV	N	6.0	Y	Y	N	N	N	N	100	100	GTR	N
42	IV	Y	5.6	Y	Y	N	N	N	N	90	90	GTR	N
43	IV	N	4.5	Y	Y	Y	N	N	N	50	50	GTR	N
44	IV	Y	4.9	Y	Y	N	P	N	N	90	80	GTR	N
45	IV	Y	5.0	Y	Y	N	N	N	N	90	90	GTR	N
46	IV	N	4.5	Y	Y	Y	T	N	N	80	70	STR	N
47	II	N	2.0	Y	N	N	T	N	N	100	80	STR	N

Patient characteristics of the 47 patients, which underwent surgical resection. Tumor diameter (in cm), preoperative deficit, postoperative deficit (T = temporary, P = permanent, N = no deficit), and Karnofsky Performance Scale (KPS) are outlined. Y = yes, N = no. TMZ = Temozolomide. RTx = radiotherapy. EOR = extent of resection. STR = subtotal resection. GTR = gross total resection.

Twenty-nine patients (62%) underwent surgery for recurrent gliomas (grade II: 3 cases; grade III: 9 cases; grade IV: 17 cases). Most common initial symptoms of the patients were seizures in 22, paresis in 13, aphasia in 4, and hemihypesthesia in 2 cases.

### Preoperative evaluation

All patients underwent preoperative magnetic resonance imaging (MRI) for tumor diagnosis, localization, preoperative assessment, and for intraoperative neuronavigation (BrainLAB Vector Vision 2® and BrainLAB Curve, BrainLAB AG, Feldkirchen, Germany). Moreover, all patients also received postoperative MR imaging to evaluate the extent of the resection. In addition, every patient was thoroughly examined before and after surgery according to a standardized protocol including handedness, muscle strength, coordination, sensory evaluation, and cranial nerve function. Muscle strength was graded for every muscle in accordance with the British Medical Research Council Scale (BMRC) preoperatively, on the first postoperative day, on the day of discharge, and during postoperative follow-up. Language function was assessed by the Aachen Aphasia Testing Battery preoperatively, at the fifth postoperative day, and 3 and 6 months after surgery [24].

The decision for the use of the different intraoperative techniques such as ultrasound, neuronavigation, fiber tracking, MEP monitoring, or awake surgery was done by the operating surgeon depending on the specific tumor location.

### Anesthesia

As volatile anesthetics have been shown to severely interfere with IOM, we used total intravenous anesthesia in all cases without exception and strictly avoided the use of volatile anesthetics before and during surgery [25-27]. Thus, anesthesia was induced and maintained by continuous propofol administration, and intraoperative analgesia was achieved through continuous administration of remifentanyl. Neuromuscular blocking was avoided during surgery and only used for intubation by rocuronium.

### Neuronavigation

Positron emission tomography (PET) images were fused with continuous sagittal images of T1-weighted 3D gradient echo sequence, T2 FLAIR, and DTI data. In 11 patients (23%), nTMS was also used to map cortical language and motor areas preoperatively; nTMS data were then fused into the neuronavigation dataset. Finally, data were transmitted to the neuronavigation system (BrainLAB Vector Vision 2® and BrainLAB Curve®, BrainLAB AG, Feldkirchen, Germany), as previously described [13,14].

### Intraoperative MEP monitoring

IOM by direct cortical stimulation was used in 38 of 47 cases (81%). Subsequent to craniotomy and durotomy, a strip electrode with eight contacts (ADTech® strip electrode, AD Technic, City, WI, USA or Inomed Medizintechnik, Emmendingen, Germany) was positioned subdurally onto the cortex of the rolandic region. An angle of 60 − 70° to the supposed central sulcus was aimed at. After positioning the strip electrode, the median nerve was stimulated and the central sulcus was identified by somatosensory evoked potential phase reversal [28]. DCS mapping of the motor cortex was then performed with intensities between 5 and 14 mA, square-wave pulse with duration of 0.2 – 0.3 ms, frequency of 350 Hz, and a train of 5 pulses as previously reported [15,28,29]. To stimulate motor evoked potential (MEP) monitoring of the upper and lower extremity, square-wave pulses with duration of 200–700 μs, a frequency of 350 Hz, and a train of 5 pulses were applied. The used protocol was published previously [15]. Decline in amplitude of more than 50%, which was not explained by technical issues, was considered a considerable deterioration and was reported to the surgeon. If changes of compound muscle action potential (CMAP) occurred, the event was instantly reported to the neurosurgeon, who reversed the causative maneuver, if possible. Partial loss of CMAP from related muscle groups was regarded as a decline rather than a loss. Latency increases devoid of concomitant deterioration of amplitude rarely occurred.

### Awake monitoring

Awake surgery was only performed when the tumor was within the left insula, operculum, dorsal superior temporal gyrus, angular gyrus, and supramarginal gyrus. Tumors within the left pre- or postcentral gyrus were not operated by awake surgery. The day before surgery, a neuropsychologist trained the patient for the object naming task and baseline testing of all pictures was performed. Only pictures that were named fluently were included for intraoperative mapping. In surgery, the patient was positioned supine and 45° to the right side. Before sharp fixation of the head, regional anesthesia was applied to the galea by bupivacaine. Fifteen minutes before language mapping, propofol infusion was stopped and remifentanyl was progressively reduced to achieve an optimum level of analgesia during mapping. DCS mapping was performed using bipolar stimulation every 5 mm using 3 to 15 mA over 4 seconds and a 60 Hz technique. To detect afterdischarges, a direct cortical electroencephalogram was recorded with 8 channels. During mapping, pictures of common objects were presented to the patient in a time-locked way, and elicited speech impairment was evaluated by the neuropsychologist. The patient had to name the object and start every naming with the sentence “This is…” Positive sites were marked at the cortical surface with numbers indicating the evoked disturbance. After completion of cortical mapping, the resection was performed under continuous language testing to also monitor affection of subcortical fiber tracts. After resection, the patient was then sedated during wound closure.

### Tumor resection

An ultrasound aspirator (Sonopet Ultrasonic Aspirator, Stryker Medical, Portage, MI, USA) as well as neuronavigation was used for all cases. Upon any amplitude loss or decline of more than 50% of the initial MEP amplitude in at least one channel, resection was halted, spatulas removed, and the surgical field was irrigated with warm Ringer’s solution. The MEP technique is extensively described above (Intraoperative MEP monitoring). In cases of awake surgery, resection was immediately stopped whenever the neuropsychologist reported deterioration of language function. In cases of resection close to a major vasculature, the surgical field was irrigated with nimodipine to reverse potential vasospasm. After renormalization/stabilization of MEPs, resection was continued. If potentials did not recover, resection was stopped at this tumor region.

### Postoperative evaluation

For every patient, neurological status was directly assessed after surgery, 6–8 weeks postoperatively and during follow-up on a regular basis every 3–12 months, depending on the tumor entity. Moreover, each patient underwent an MRI scan within 48 hours after operation. During follow-up, MRI scans were also performed every 3–12 months depending on the tumor grade. Thus, we evaluated the MRI scan of the first postoperative day with regard to the extent of the resection, increasing edema, diffusion impairment, and bleeding to find explanations for neurological deterioration without intraoperatively MEP changes. Extent of resection was defined as gross total resection (GTR) or subtotal resection depending on the presence of residual tumor on T2 FLAIR (WHO grade II and III) or T1 contrast-enhanced sequences (WHO grade I and IV). Furthermore, we evaluated every MRI scan during follow-up for recurrent tumors. Neurological status in this study was only considered during progression-free survival. New postoperative neurological motor deficit was distinguished between temporary and permanent deficit. Temporary deficit was defined as a new or aggravated postoperative motor deficit that disappeared at least until the 6- to 8-week follow-up. Permanent deficit was defined as new or aggravated postoperative motor deficit that did not resolve during follow-up.

### Ethical standard

The study is well in accordance with the ethical standards of the Technical University of Munich, the local ethics committee (registration number: 2826/10), and the Declaration of Helsinki.

### Statistical analysis

To test the distribution of several attributes, a chi-square or Fisher Exact test was performed. Differences between groups were tested using the Kruskall-Wallis test for nonparametric one-way analysis of variance (ANOVA), followed by Dunn’s test as the post hoc test. Differences between two groups were tested using the Mann–Whitney-Wilcoxon test for multiple comparisons on ranks for independent samples, followed by Dunn’s test as the post hoc test. All results are presented as mean ± standard deviation (SD). Median and range were also delivered (GraphPad Prism 5.0 c, La Jolla, CA, USA); p < 0.05 was considered significant.

## Results

**Figure 1:**
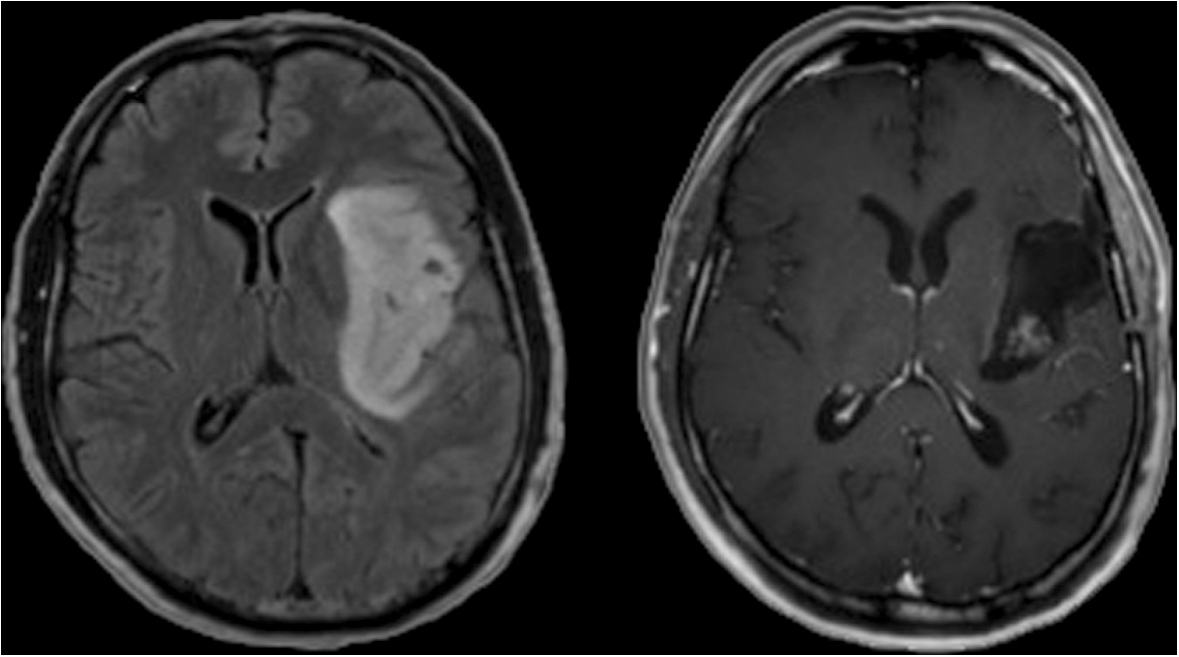
Illustrative case of gross total resection of a left-sided insular glioma WHO grade 3.

GTR was achieved in 35 cases (74%) (Figure 1). Awake surgery was performed in 8 cases (17%), whereas 38 cases (81%) were performed under continuous MEP monitoring. Three cases (6%) received awake craniotomy and MEP monitoring for subcortical dissection within the pyramidal tract after the awake phase. Thus, 4 cases underwent surgery without MEP or awake monitoring. For evaluation and follow-up of neurological function, we only considered neurological status during progression-free survival, which was 11.3 months (range: 2 weeks – 64.5 months) and median overall survival was 14.8 months (range: 4 weeks – 20.5 months) depending on recurrence and malignancy (Table 2). Before surgery, not only recurrent but also some newly diagnosed gliomas were treated using chemo- or radiotherapy. Table 3 provides an overview. Moreover, there were no healing problems or postoperative infections in the patients within this cohort.

**Table 2:** Follow-up and overall survival

	**mean follow-up (months)**		**mean overall survival (months)**	
	**primary**	**recurrent**	**primary**	**recurrent**
WHO grade I	47.9	-	alive	-
WHO grade II	38.3	26.0	all alive	all alive
WHO grade III	21.6	22.0	all alive	20.5
WHO grade IV	8.6	7.9	5.1	6.0

Columns 2 & 3: mean follow-up for alive patients. Columns 4 & 5: overall survival of deceased patients. This series only contains one patient with initially diagnosed WHO grade I glioma. When patients are alive, mean overall survival equals to mean follow-up.

**Table 3:** Presurgical therapy

	**Primary surgery**		**Recurrent tumor**	
	**cases**	**%**	**cases**	**%**
RTx only	2	11	0	0
TMZ only	6	33	8	28
TMZ + RTx	0	0	2	7
Seed	0	0	1	3

An overview on presurgical chemo- or radiotherapy in patients with recurrent but also with initially diagnosed gliomas, after which non-resectability was noted. Temozolomide (TMZ) and radiotherapy (RTx) were also applied combined.

### Preoperative functional mapping

Navigated TMS was used for preoperative mapping of language areas in 4 cases and motor areas in 6 cases because 2 cases underwent combined motor and language mapping.

### Further used modalities

Neuronavigation was applied in all cases. Diffusion tensor imaging fiber tracking was included in 18 (38%); fluorescence guidance using 5-aminolevulinic acid was applied in 18 (38%); and intraoperative ultrasound was used in 1 case.

### Awake craniotomy

Of patients undergoing awake surgery, 5 patients (63%) suffered from initially diagnosed and 3 patients (37%) suffered from recurrent glioma. After awake craniotomy on 8 patients, 6 patients (75%) showed a new aphasia at the first postoperative day but only 1 patient (13%) experienced a permanent surgery-related aggravated aphasia during long-term follow-up. GTR was possible in 5 cases (63%).

### Correlation of tumor type and location to postoperative motor deficit

**Figure 2:**
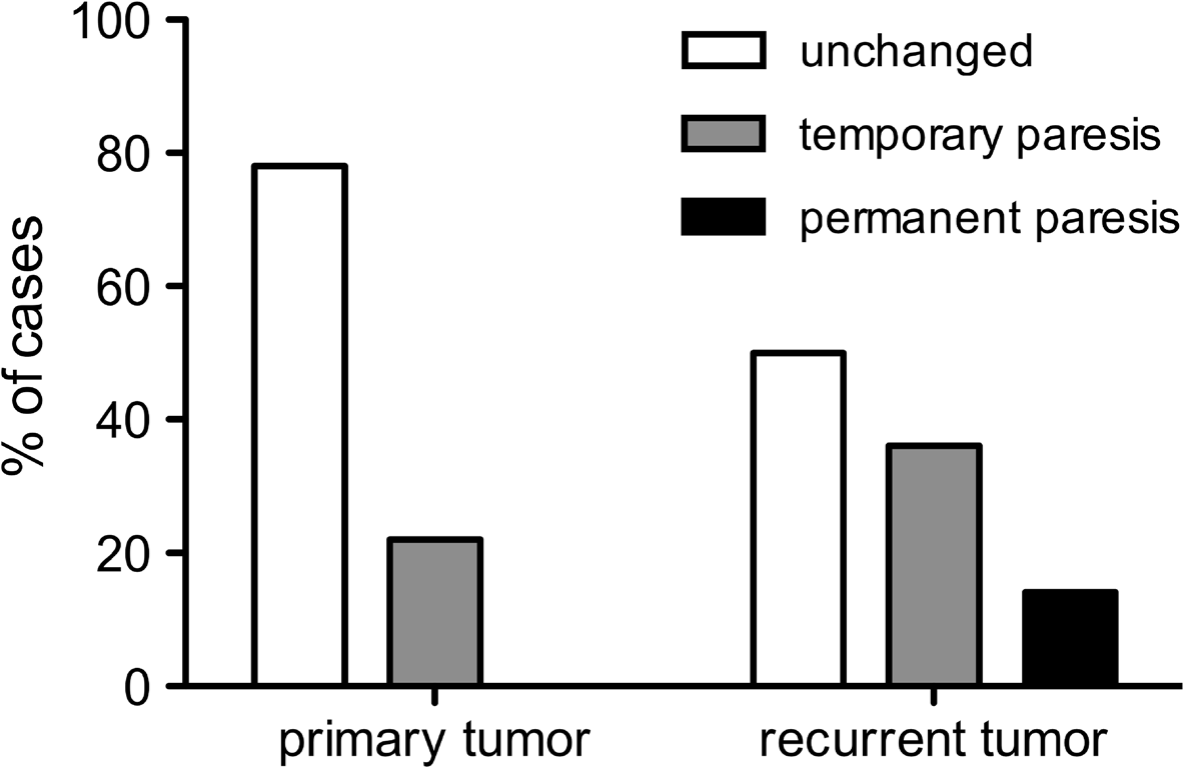
**Recurrent glioma.** Postoperative impairment of motor function is higher after resection of recurrent tumors compared to gliomas undergoing initial resection (p < 0.01185).

Postoperative temporary or permanent impairment of motor function was significantly higher in recurrent tumors: After primary glioma resection (18 patients), no patients showed any permanent deficit, whereas 4 patients (22%) presented with temporary and 14 patients (78%) with no new postoperative motor deficit. However, after resection of recurrent glioma (28 patients), 4 patients (14%) showed permanent and 10 patients (34%) showed temporary surgery-related new paresis. Thus, 15 patients (52%) showed no new motor deficit (Figure 2). As expected, postoperative temporary and permanent impairment of motor function were related to tumor location with no respect to initial or recurrent tumor. After resection of gliomas in the precentral gyrus, 11% of all patients (1 patients) experienced permanent deterioration of motor function. Additionally, 44% of patients (4 patients) with a precentral glioma showed a temporary motor function deficit. After resection of insular gliomas, patients showed temporary deficit in 33% (5 patients) and permanent deficit in 7% of all cases (1 patient). Patients with gliomas affecting the subcortical white matter temporarily deteriorated in 67% (2 patients) and permanently deteriorated in 33% (1 patient) of cases with regard to motor function.

### MEP monitoring

**Figure 3:**
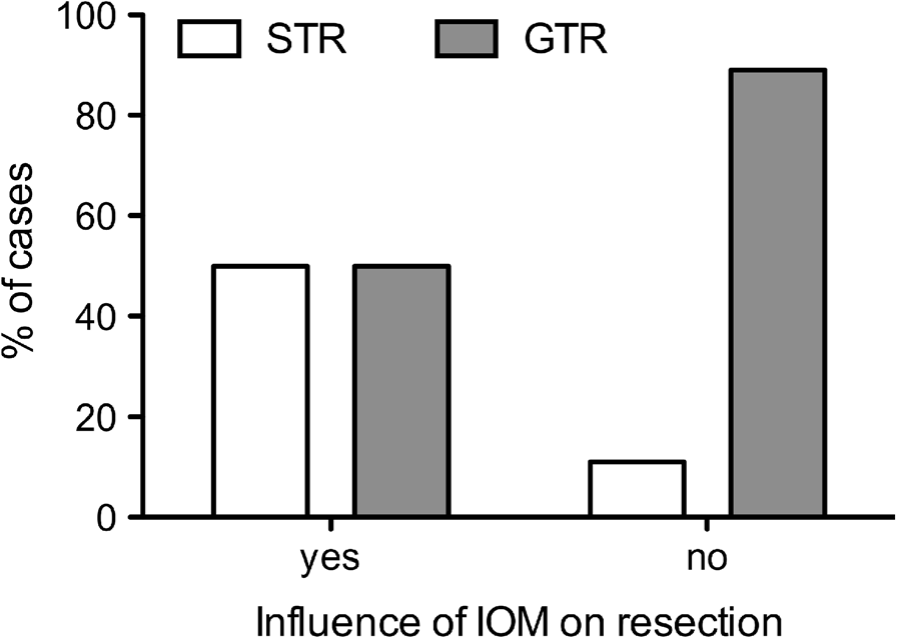
**Influence of IOM on the extent of resection.** When surgery was influenced by IOM due to MEP amplitude decline of more than 50% baseline, gross total resection (GTR) was only achieved in 50% of cases, whereas GTR was achieved in 89% of cases in which IOM showed no impact on surgery due to stable amplitudes (p0.0186).

**Figure 4:**
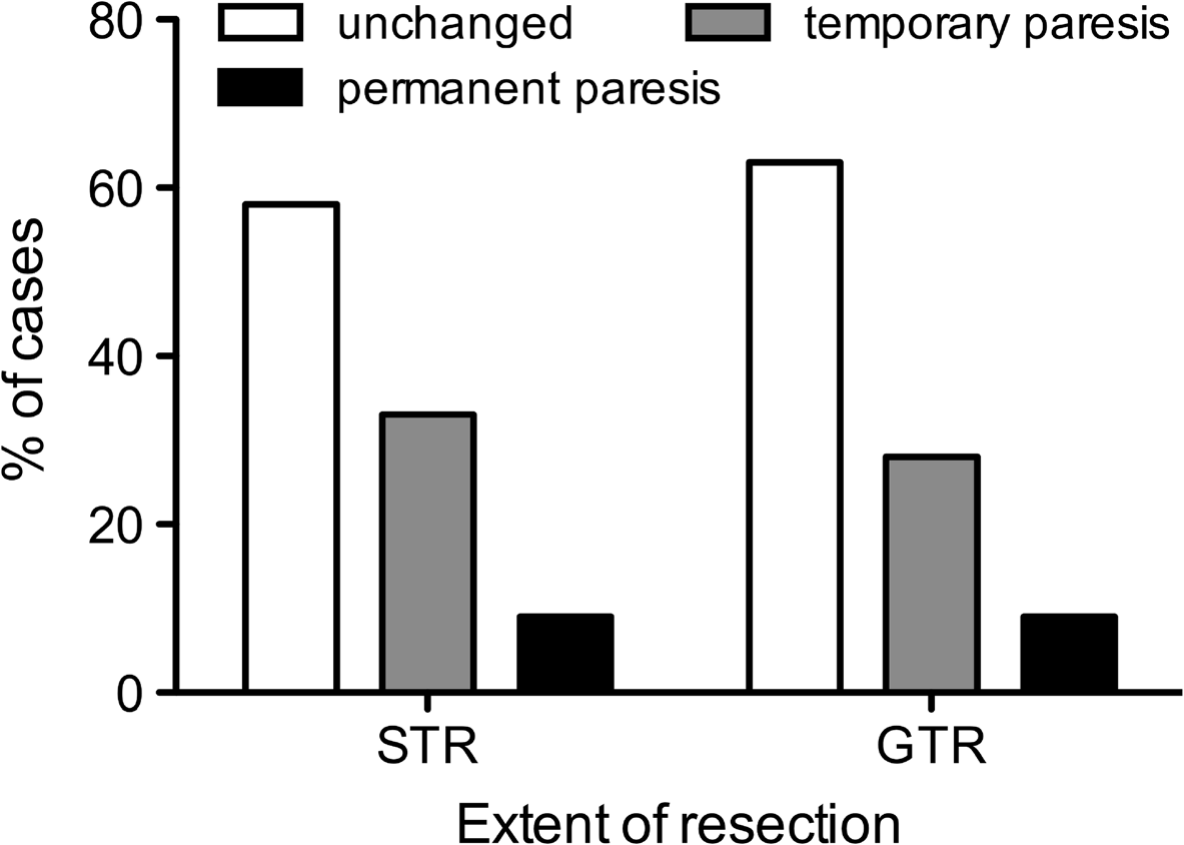
**Extent of resection vs. postoperative paresis.** Postoperative new temporary or permanent motor deficits were highly comparable in patients with subtotal (STR) and gross total (GTR) resection.

**Figure 5:**
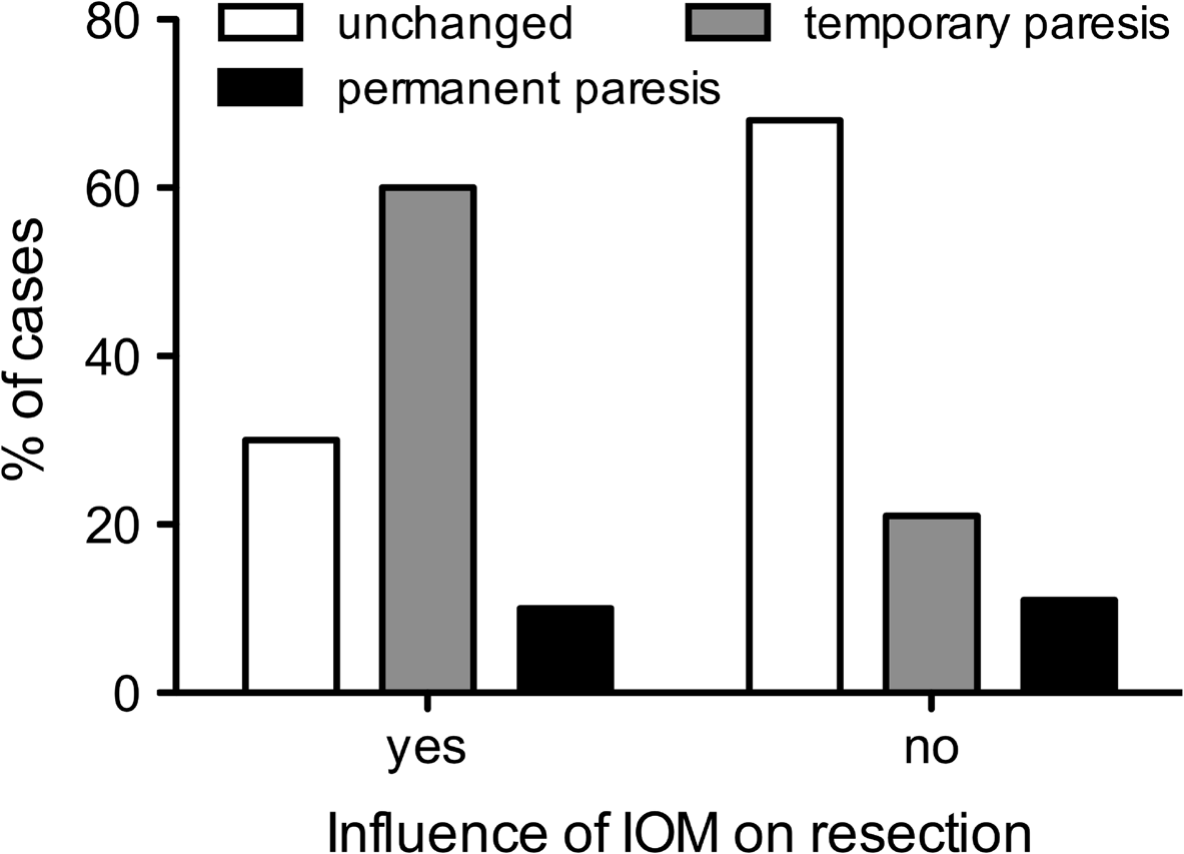
**IOM vs. postoperative paresis.** When surgery was influenced by IOM due to MEP amplitude decline of more than 50% baseline data showed a trend towards a higher rate of temporary motor deficit compared to patients in which resection was not affected by IOM (p = 0.07).

In all intended 38 cases, IOM through continuous MEP monitoring was possible. MEPs were stable throughout the operation in 18 patients (47%), showed reversible amplitude decline of more than 50% baseline but recovered in 15 patients (39%), and irreversible amplitude declined more than 50% baseline in 5 patients (13%). Postoperatively, 18 patients (39%) had a new motor deficit, which remained permanent in 4 patients (8.5%). Irreversible MEP decline was only observed in WHO grade III and grade IV gliomas, but no other significant difference existed with respect to the different tumor types (data not shown). Out of those 20 cases (52%) with MEP amplitude decline, resection was temporarily stopped, attributable to IOM in 10 cases (26% of all 38 IOM cases) and completely halted in 6 of these cases (16% of all 38 IOM cases). Immediately after MEP decline, retractors were repositioned and the resection cavity was additionally irrigated. In 5 of these 10 cases (50%), STR was achieved, whereas STR was performed in only 3 out of 28 cases (11%), which were not influenced by IOM due to stable amplitudes (p = 0.0186; Figure 3). Postoperative new temporary or permanent motor deficits were similar in the STR (unchanged: 58%, temporary: 33%, permanent: 9% of 12 cases) and GTR groups (unchanged: 63%, temporary: 28%, permanent: 9% of 35 cases) (Figure 4). In contrast, in those 10 cases in which the surgeon had to stop resection because of considerable MEP decline, we recognized an unchanged motor function in 30% of cases and a new temporary deficit in 60% of cases, and new permanent motor deficit in 10% of cases. Without the influence of IOM, motor function was unchanged in 68% of cases, temporarily deteriorated in 21% of cases, and permanently deteriorated in 11% of cases (p = 0.07; Figure 5). Although the data failed to show statistical significance, they showed a trend toward a higher rate of temporary motor deficit in patients in which resection was limited by IOM.

### Postoperative MRI scans

To find sufficient basis for the explanation of postoperative neurological deterioration, we evaluated all postoperative MRI scans. Nine patients (13%) had temporary new motor deficit despite recovered MEP decline in which MRI revealed increasing edema in 4 cases and secondary hemorrhage within the resection cavity in 5 cases. However, only 3 out of these 5 cases were symptomatic and underwent revision surgery at the same day. Out of those 4 patients with new permanent surgery-related paresis, 2 presented with ischemic lesions at the border of the resection cavity and 2 showed resection within motor eloquent regions. With regard to the 8 awake cases, 2 patients showed temporarily and 1 patient presented with permanently deteriorated language function. All 3 cases were glioblastoma multiforme within the angular gyrus and postoperative MRI showed no edema, hemorrhage, or ischemia.

### Operation on recurrent gliomas

In this series, we operated on 29 recurrent gliomas. Three were WHO grade II, 9 were WHO grade III, and 17 were glioblastoma (GBM). Of these patients, 7 (24%) already had preoperative paresis. Four patients were operated awake and one of these patients (25%) suffered from preoperative aphasia. However, continuous MEP monitoring was possible in all 24 intended cases (83%). Compared with the first operation, resection of recurrent gliomas showed a lower degree of subtotal resections but without reaching statistical significance (17% in recurrent and 39% in the first operation). Concerning resections of recurrent glioma, postoperative new permanent deficits were observed in 14% of all cases (4 patients) (aphasia: 3%, paresis: 11%), whereas temporary deficits occurred in 35% of cases (10 cases) (aphasia: 10%, paresis: 25%) (Figure 2). Pre- as well as postoperative KPS was also comparable in patients who underwent the first (before: 85, after surgery: 90) and repeated resection (before: 85, after surgery: 80).

## Discussion

During the last decade, surgical resection became increasingly important as part of a multimodal therapeutic regime for the treatment of high- and low-grade gliomas [1,4,23,30,31]. However, even today, many gliomas within highly eloquent cortical regions still regularly undergo only debulking or biopsy. The most striking argument for this approach is the risk of surgery-related deficits [6-9]. Nonetheless, with regard to already published data on surgery on eloquent gliomas, the risk of new neurological deficits seems moderate [15,18,22,23,31-33]. Especially when a multimodal and function-guided approach is used [34]. Yet, no studies or subgroup analyses exist that reviewed the actual functional outcomes and oncological benefits of surgery in patients initially diagnosed as inoperable.

In our series, only 8.5% of all patients with gliomas in or adjacent to eloquent motor areas suffered from new permanent deterioration of motor function after surgery (Figure 2). Regarding these data, our study is well in accordance with previous studies [26,35,36]. When also considering the high postoperative KPS in initially diagnosed and recurrent gliomas, we have to strongly reject the argument that these patients have an unacceptable high risk of surgery-related disability or loss in quality of life.

With regard to the GBM subgroup, median survival was comparable to the non-surgical series; however, KPS was higher in our patients even after surgery. Thus, high-quality survival was improved (Table 2) [7-9,37].

Concerning the impact of the extent of resection on the actual survival the subgroups of this study are to small for such a statistical analysis. Thus, this study has to be considered as a pilot study.

### Preoperative functional mapping

As also reported previously, we observed an important impact from preoperative nTMS mapping of the motor eloquent cortex [14,38]. Moreover, 4 additional patients underwent nTMS mapping of the language eloquent cortex. Although nTMS language mapping still requires further research, it is already a valuable tool in a multimodal approach [39,40].

### Correlation of tumor type and location to postoperative motor deficit

In our series, most tumors were located within the insula, rolandic region, or the perisylvian cortex. When analyzing our data, we were not able to show any statistically significant difference for the risk of surgery-related new motor deficit with regard to tumor location. Thus, we cannot identify any of these structures to be less eligible for surgical resection, which is well in accordance with previous findings [15]. However, we must emphasize that surgery of recurrent glioma has a significantly higher risk of surgery-related new motor deficit (Figure 2), which was also found by others and has to be kept in mind when advising our patients [15,41]. The reasons for this phenomenon are supposed to be primarily vascular. As primary resection of these gliomas usually reaches the borders of motor or language eloquent regions, recurrent tumor growth invades this eloquent brain tissue and its supplying arteries. Thus, our series showed that surgery of recurrent gliomas causes a higher rate of ischemia adjacent to the resection cavity as initial surgery does, which is contradictive to previous studies [41]. Moreover, chemotherapy as well as radiation therapy might alter neuronal and vascular metabolism and therefore impair motor plasticity as it has been described recently [42].

### MEP monitoring

MEP amplitude decline caused a significantly higher rate of STR (Figure 3). However, this group also showed a lower rate of temporary but not of permanent new motor deficits (Figure 4). However, this result seems to mostly come from the small number of cases (10 patients) in which surgery was influenced by IOM. Without the influence of IOM on motor function, we failed to show statistical significance. However, the data showed a trend toward a higher rate of temporary motor deficit in patients in which resection was limited by IOM. Yet, the rate of permanent motor deficits was identical (Figure 5). These findings have to be interpreted as a result of the small group of patients with influence of IOM on the course of surgery (10 patients) because larger series indeed showed an influence of IOM on the functional outcome of long-term follow-up [15,17].

Concerning those 2 cases of reversible MEP decline with permanently new motor deficit, in which we observed partial removal of the primary motor cortex we have to state that this partial resection of rolandic cortex is not the only explanation although it is the only explanation, which can be observed on postoperative MRI. A dislocation of the cortical MEP electrode and replacement to another cortical muscle representation is also an explanation that has to be mentioned.

### Recurrent gliomas

In this series, we operated on 29 recurrent gliomas. Compared with the first operation, resection of recurrent gliomas showed a surprisingly lower degree of STR but without reaching statistical significance (17% in recurrent and 39% in the first operation). However, a higher rate of very relevant postoperatively new permanent deficits was observed (aphasia: 3%; paresis: 11%; see Figure 2). Nonetheless, pre and postoperative KPS was also comparable in patients who underwent the first and repeated resection, which shows a persistent quality of life. In particular, our data on potential survival rates offers further evidence that reoperation of recurrent high-grade gliomas is beneficial. Although some authors stated that a second surgery for high-grade gliomas is comparable to conservative treatment [43], others provided evidence that surgery improves survival and quality of life in most patients [44].

Moreover, as mentioned in Table 3, only 2 patients with recurrent gliomas underwent both chemo- and radiotherapy as initial treatment. With regard to the supposed standardization of glioma therapy, this number is rather small and shows us that even more standardization or even centralized and not only interdisciplinary neuro-oncological tumor conferences might be indicated.

## Conclusions

Our results showed that gliomas judged as non-resectable are potentially eligible for surgical resection. By using a multimodal approach including preoperative functional mapping, IOM, and awake craniotomy in some cases, achieving a high extent of resection at an acceptable rate of postoperative neurological deterioration is possible. Particularly after primary resection, no patient in our series suffered from any new permanent deficit. With regard to this data, patients with primarily rated “inoperable” gliomas should be referred to a specialized center to achieve the best oncological basis by surgical resection for an adjuvant therapy. Although the rate of new surgery-related neurological deficits is low and postoperative KPS and survival advocates for a surgical approach in the vast majority of cases, this decision must be discussed individually with every patient and in the context of a neuro-oncological conference including neurosurgical, neurologists, neuroradiologists, and radiotherapist. Moreover, neurosurgical centers with limited expertise on surgery of such highly eloquent lesions should strongly refer their patients for a second opinion to a specialized center.

## Abbreviations

ANOVA: nonparametric one-way analysis of variance; BMRC: British Medical Research Council Scale; CMAP: compound muscle action potential; DCS: direct cortical stimulation; GBM: glioblastoma; GTR: gross total resection; IOM: intraoperative neuromonitoring; KPS: Karnofsky performance scale; MEP: motor evoked potentials; MRI: magnetic resonance imaging; nTMS: navigated transcranial magnetic stimulation; PET: positron emission tomography; SD: standard deviation; STR: subtotal resection.

## Competing interests

The authors declare that they have no conflict of interest that affects this study. The study was completely financed by institutional grants from the Department of Neurosurgery. The authors report no conflict of interest concerning the materials or methods used in this study or the findings specified in this paper.

## Authors’ contributions

SK was responsible for data acquisition, handled the acquired data and performed literature research as well as statistical analyses. SK drafted the manuscript and its final revision. SK is also responsible for concept and design. LS was responsible for data acquisition, performed data analysis and clinical assessment. ES was responsible for data acquisition and approved and corrected the final version of the manuscript. DD was responsible for data acquisition, read and approved the final manuscript. TO and NB were responsible for data acquisition and approved and corrected the final version of the manuscript. JG and BM approved and corrected the final version of the manuscript. FR is responsible for the original idea, the concept, design, and statistical analyses. FR has also written and revised the manuscript, approved and corrected the final version. All authors read and approved the final manuscript.

## Authors’ information

All authors are strongly involved in the treatment of brain tumors including awake surgery, preoperative mapping, and intraoperative neuromonitoring in a specialized neurooncological center. BM is chairman and FR is vice chairman of the department.

## Pre-publication history

The pre-publication history for this paper can be accessed here:

http://www.biomedcentral.com/1471-2407/13/51/prepub
